# Advanced Care Provider and Nursing Approach to Assessment and Management of Immunotherapy-Related Dermatologic Adverse Events

**DOI:** 10.6004/jadpro.2017.8.2.2

**Published:** 2017-03-01

**Authors:** Kathryn Ciccolini, Anna Skripnik Lucas, Alyona Weinstein, Mario Lacouture

**Affiliations:** 1 Dermatology Service, Memorial Sloan Kettering Cancer Center, New York, New York;; 2 Melanoma and Immunotherapeutics Service, Memorial Sloan Kettering Cancer Center, New York, New York

## Abstract

Advanced care providers (ACPs) and nurses are fundamental players in the assessment and management of immunotherapy-related dermatologic adverse events (irdAE). Pembrolizumab, nivolumab, and ipilimumab are approved for unresectable or metastatic melanoma, metastatic non–small cell lung cancer (pembrolizumab and nivolumab), metastatic head and neck squamous cell carcinoma (pembrolizumab and nivolumab), advanced renal cell carcinoma, and Hodgkin lymphoma (nivolumab). Atezolizumab is approved for urothelial carcinoma. These agents function as immune checkpoint inhibitors, activating T-cell–mediated antitumor immune responses through the inhibition of the programmed cell death protein 1 (PD-1) or cytotoxic T-lymphocyte antigen 4 (CTLA-4). Immune checkpoint inhibitors have been reported to cause irdAEs, including rash, pruritus, and vitiligo, requiring an interdisciplinary approach to avoid dose reduction or discontinuation of treatment and to maintain quality of life. Advanced care providers and nurses play a critical role in the attribution, grading, and management of these untoward events and must be knowledgeable about their pathophysiology, incidence, assessment, and clinical presentation.

Through recent research, tumors have demonstrated the ability to shield the normal immune response by exploiting immune checkpoint pathways (Harvey, 2014). Two major immune checkpoint pathways that are being researched are cytotoxic T-lymphocyte antigen 4 (CTLA-4) and programmed cell death protein 1 (PD-1; Pardoll, 2012). The CTLA-4 protein receptor is expressed on T cells, which downregulate the immune system (Lacouture et al., 2014; Melero, Hervas-Stubbs, Glennie, Pardoll, & Chen, 2007; Seetharamu, Budman, & Sullivan, 2016). The PD-1 receptor found on T cells binds to programmed cell death ligand 1 or 2 (PD-L1/PD-L2), resulting in T-cell deactivation and negative immune response regulation (Pardoll, 2012).

## BACKGROUND ON IMMUNE CHECKPOINT INHIBITORS

Ipilimumab (Yervoy) was approved in 2011 for the treatment of unresectable or metastatic melanoma and was the first agent to demonstrate a survival benefit in the treatment of metastatic melanoma (Hodi et al., 2010; Khoja et al., 2016). It is administered intravenously at 3 mg/kg over 90 minutes every 3 weeks, for a total of 4 doses. Ipilimumab is a fully human monoclonal antibody designed to promote antitumor immunity by inhibiting CTLA-4 and CD80/CD86 ligands, resulting in T-cell activation and proliferation (Bristol-Myers Squibb [BMS], 2011).

Pembrolizumab (Keytruda) was granted approval in 2014 for unresectable or metastatic melanoma and disease progression following ipilimumab and if found to have *BRAF* V600 mutation–positive disease, then a BRAF inhibitor is added to the regimen. The US Food and Drug Administration (FDA)-approved dose is an intravenous infusion of 2 mg/kg over 30 minutes every 3 weeks (Merck, 2016; Pazdur, 2014). Pembrolizumab is also approved for patients with recurrent or metastatic head and neck squamous cell carcinoma with disease progression on or after platinum-containing chemotherapy. The dose approved for this patient population is 200 mg every 3 weeks (Merck, 2016).

Nivolumab (Opdivo) received expedited approval in 2014 for the treatment of *BRAF* V600 wild-type unresectable or metastatic melanoma as a single agent or in combination with ipilimumab. It is also FDA approved for *BRAF* V600 mutation–positive unresectable or metastatic melanoma, metastatic non–small cell lung cancer (NSCLC) and disease progression on or after platinum-based therapy, advanced renal cell carcinoma (RCC) after receiving prior antiangiogenic therapy, classical Hodgkin lymphoma (HL) that has relapsed or progressed after autologous hematopoietic stem cell transplant and posttransplantation brentuximab vedotin (Adcetris), and recurrent or metastatic squamous cell carcinoma of the head and neck (SCCHN) with disease progression on or after a platinum-based therapy. The FDA-approved dose for unresectable or metastatic melanoma is 240 mg every 2 weeks (single agent), 1 mg/kg followed by ipilimumab on the same day every 3 weeks for 4 doses, and then nivolumab at 240 mg every 2 weeks. For metastatic NSCLC, SCCHN, HL, and advanced RCC, the dosing is 3 mg/kg every 2 weeks (BMS, 2016).

Atezolizumab (Tecentriq) was approved in 2016 for the treatment of patients with locally advanced or metastatic urothelial carcinoma who have disease progression during or after platinum-containing chemotherapy or disease progression within 12 months of neoadjuvant/adjuvant treatment with platinum-containing chemotherapy. The FDA-approved dosing is 1,200 mg as an intravenous infusion over 60 minutes every 3 weeks (Genentech, 2016).

Pembrolizumab and nivolumab are anti–PD-1 agents, and atezolizumab is an anti–PD-L1 agent. Together, they mediate a T-cell response, resulting in antitumor suppression (Genentech, 2016; Merck, 2016). In various cancers, such as melanoma, PD-L1 can be expressed on the tumor cell surface and can bind to the T-cell receptor PD-1 to manipulate the immune checkpoint pathway, suppressing T-cell tumor attack and promoting tumor growth advantage (Grosso et al., 2013; Hamid et al., 2013). These highly selective human monoclonal antibodies restore and potentiate T-cell antitumor response by inhibiting the nexus of PD-1 on T cells and PD-L1 expression on tumor cells—the PD-1 blockade pathway (Hamid et al., 2013; Harvey, 2014; Menzies & Long, 2013). The combination of ipilimumab and nivolumab has also been approved for the treatment of advanced melanoma (Larkin et al., 2015).

Dermatologic conditions in an oncology setting have been reported to cause a negative impact on quality of life (Gandhi, Oishi, Zubal, & Lacouture, 2010; Rosen et al., 2013). Importantly, the trajectory of these untoward events may ultimately lead to inconsistent dosing and discontinuation of therapy, which may affect clinical outcomes (Lacouture et al., 2011). The study of immunotherapy-related dermatologic adverse events (irdAEs) underscores the field of supportive oncology in addressing untoward events, quality of life, and psychosocial impact. The purpose of this article is to present advanced care providers and nurses with an overview of pathophysiology, incidence, assessment, and clinical presentation of unresectable/metastatic melanoma patients treated with immunotherapy and to aid in the management of the irdAE.

## PATHOPHYSIOLOGY

Immunotherapy-related dermatologic adverse events are thought to be caused by the imbalance of immune checkpoint response. The pathophysiology of rashes induced by anti–PD-1 remains unclear. However, Belum et al. (2016) suggested a reflection of onset and pattern seen with CTLA-4 inhibitors such as ipilimumab, with histology representing a lichenoid tissue reaction/interface dermatitis. Furthermore, Naidoo et al. (2015) noted the histology of these rashes has revealed an "interface, perivascular, and periadnexal lymphocytic dermatitis, with few plasma cells and eosinophils" (p 2376). For rashes associated with CTLA-4 inhibition with ipilimumab, histology has demonstrated perivascular immune cell infiltrates in superficial dermis that extends to the epidermis in both lymphocytic and eosinophilic infiltrates. In addition, melan-A–specific CD8-positive T cells have been noted with ipilimumab rash infiltration (Lacouture et al., 2014). Lastly, Jaber et al. (2006) conducted a single-institution prospective study and reported that in 6 of 9 patients treated with ipilimumab at a starting dose of either 3 mg/kg or 5 mg/kg, eosinophilia was observed at the time of skin eruptions (p = .006).

Pruritus with anti–CTLA-4 therapy has been described to be directly associated to the inhibition of the receptor and the enhancement and activation of the immune system in the skin (Fischer, Rosen, Ensslin, Wu, & Lacouture, 2013). Histologic manifestations of skin reactions in stage IV melanoma with ipilimumab therapy have been reported to be superficial; perivascular CD4-positive predominant T-cell infiltrate with eosinophils in the dermis and mild epidermal spongiosis were present (Jaber et al., 2006; Lacouture et al., 2014).

Byrne and Turk (2011) reported the dualistic pathogenesis of vitiligo; antibody (tyrosinase-related protein 1 and 2 [TRP-1/TRP-2]) vs. T-cell related (CD8-positive). The authors noted the pathogenesis of CTLA-4–induced vitiligo is not well understood, and further investigation is needed. Vitiligo and vitiligo-like depigmentation is an autoimmune response that can occur with melanoma patients on immunotherapy, which results from shared expression of melanocyte-differentiation antigens (Teulings et al., 2015). Lacouture et al. (2014) explained CTLA-4 inhibition and immune system activation can directly impact vitiliginous lesions in patients receiving ipilimumab. Histologically, T cells (CD4-positive and CD8-positive) have been found next to apoptotic melanocytes, suggesting an autoimmune reaction against the melanocytes. Finally, Hua et al. (2015) noted the histology with Fontana-Masson melanin staining and anti–MART-1 (melanoma antigen recognized by T cells 1) immunohistochemistry in two patients receiving pembrolizumab revealed a dermal inflammatory infiltrate with a predominance of T cells and disappearance of skin melanocytes.

## INCIDENCE OF RASH, PRURITUS, AND VITILIGO

In 2013, Minkis, Garden, Wu, Pulitzer, and Lacouture conducted a systematic review and meta-analysis of the risk of rash associated with ipilimumab in 1,208 patients with cancer. The overall incidence of an all-grade rash was 24.3% (95% confidence interval [CI]: 21.4%–27.6%), with a relative risk [RR] of 4.00 (95% CI: 2.63–6.08, *p* < .001). The overall incidence of high-grade rash was 2.4% (95% CI: 1.1%–5.1%), with a RR of 3.31 (95% CI: 0.70–15.76, *p* = .13).

Immune checkpoint inhibitors have been reported to have an increased risk of all-grade and high-grade skin rash compared with the control group. Abdel-Rahman, ElHalawani, and Fouad (2015) calculated the RR of all-grade rash in immune checkpoint inhibitors (ipilimumab, nivolumab, tremelimumab [also known as CP-675,206], pidilizumab [formerly known as CT-011], and pembrolizumab) was 4.06 (95% CI: 3.35–4.91; *p* < .0001) and of high-grade rash was 4.81 (95% CI: 1.93–12.02; *p* = .0008). The same authors also reported the overall incidence of rash from immune checkpoint inhibitors ranged from 16% to 36%.

Belum et al. (2016)) conducted a systematic review and meta-analysis on the characterization and management of dermatologic adverse events (dAEs) to agents targeting the PD-1 receptor. The RR for generally developing a dAE was 2.95 for pembrolizumab and 2.93 for nivolumab. The incidence of all-grade rash with pembrolizumab was 16.7% (RR = 2.6) and with nivolumab, 14.3% (RR = 2.5). The incidence of all-grade pruritus with pembrolizumab was 20.2% (RR = 49.9) and with nivolumab, 13.2% (RR = 34.5). Lastly, the incidence calculated for vitiligo in patients receiving pembrolizumab was 8.3% (RR = 17.5) and with nivolumab, 7.5% (RR = 14.6). The authors concluded that pembrolizumab and nivolumab are associated with low-grade rash, pruritus, and vitiligo (Belum et al., 2016).

Interestingly, in a retrospective study by Sanlorenzo et al. (2015) studying pembrolizumab and patients experiencing dAEs with associated disease progression, a survival analysis demonstrated patients who developed dAEs had significantly longer progression-free intervals compared with those who did not develop dAEs. In addition, a retrospective analysis performed by Freeman-Keller et al. (2016) of irAEs in melanoma patients treated with nivolumab found that cutaneous irAEs were associated with melanoma survival. More controlled studies are needed to determine whether an irdAE could serve as a surrogate marker for treatment response in patients receiving anti–PD-1 inhibitors such as pembrolizumab and nivolumab.

## ASSESSMENT AND CLINICAL PRESENTATION

Advanced care providers and nurses must be skilled in both the dermatologic assessment and grading of irdAEs in patients on these novel therapies. Both clinical visits and telephone triaging are essential platforms to comprehensively assess a patient’s skin, mucosae, and associated symptoms. It is important to utilize standardized grading tools, such as the Common Terminology Criteria for Adverse Events (CTCAE) 4.0 v3, when communicating with the interdisciplinary team, including the grade and attribution (Chen et al., 2012). Both advanced care providers and nurses require sound critical judgment for expert assessment, which supports a framework for patient education and advocacy (Hickey, 2012).

**Figure 1:**
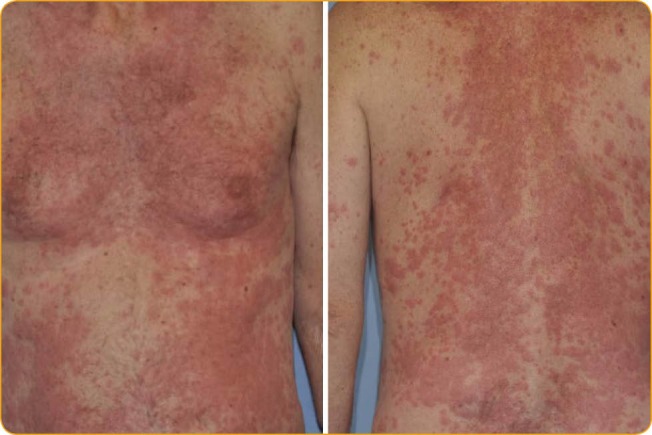
Maculopapular rash in a patient who received treatment with ipilimumab and nivolumab.

**Figure 2:**
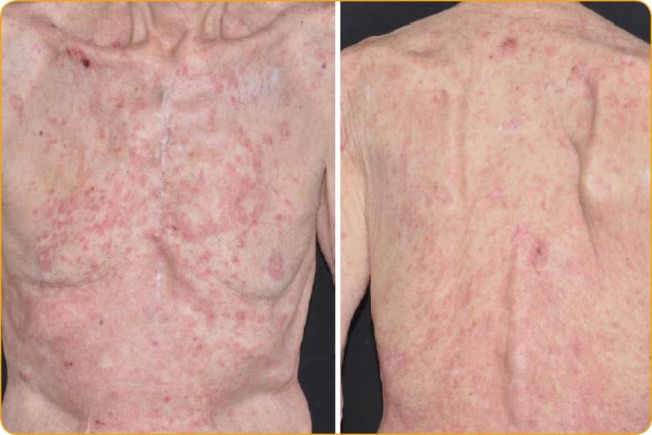
Maculopapular rash in a patient who received treatment with pembrolizumab.

Immune-related dermatologic adverse events can vary in clinical presentation, with maculopapular rash being the most typically observed variant (Figures 1 and 2), commonly presenting after the second cycle (Naidoo et al., 2015), and distribution patterns primarily seen on the extremities and trunk (Postow, Callahan, & Wolchok, 2015). Both CTLA-4 and PD-1 inhibitor–induced rashes may be associated with pruritus (Belum et al., 2016; Lacouture et al., 2014). Other types of rashes, such as lichenoid dermatitis, bullous pemphigoid, Stevens-Johnson syndrome, and toxic epidermal necrolysis, have been rarely reported with PD-1 inhibitors (Naidoo et al., 2015). For this reason, advanced care providers and nurses should perform a comprehensive history and a careful physical examination; they should also capture meticulous details of patient symptoms, risk factors, previous drug reactions, past and current medications, herbals or supplements, allergies, review of systems, and comorbidities, to identify an appropriate differential diagnosis (Bernstein, Bloomberg, Castells, Mendelson, & Weiss, 2010).

## MANAGEMENT

**Interdisciplinary Approach and Referral**

In the management of irdAEs, advanced care providers and nurses must draw upon their expert knowledge of disease process, pharmacologic mechanism of action, and the nursing process. Oncologists, dermatologists, pharmacists, nurse practitioners, physician assistants, and nurses, along with other services, are paramount in the structure of the multidisciplinary team.

There remains a dearth of research dedicated to characterizing the multifaceted approach in the prevention and management of irdAEs induced by novel agents. However, various reports on targeted therapy–induced dAEs for other malignancies demonstrate the importance of an interdisciplinary approach (Ruiz et al., 2014).

In the new era of immunotherapies, it is essential to establish advanced care provider and nursing best practices in timely referrals and coalesce a multidisciplinary approach to optimize the patient outcomes that can be achieved with novel agents (McDermott et al., 2014). Skripnik and Ciccolini (2016) have defined a nursing algorithm in the referral process for cutaneous lymphoma, which magnifies the role of the nurse in assessment, education, interdisciplinary communication, adherence to referrals, treatment, and medical recommendations. Through the use of this algorithm, advanced care providers and nurses can create a tailored referral formula for patients being referred to a dermatology specialist. A similar formula is recommended for the oncology nurse caring for patients receiving immunotherapies for appropriate screening, assessment, patient education, and advocacy, providing appropriate patient resources and ensuring the successful and timely transfer of care to a dermatology specialty. Referring patients to specialists for management is vital in streamlining patient care and optimizing providers’ expertise to improve patient outcomes.

Furthermore, the newly developed nursing CREAM principles encompass the nursing role in the Communication, Referral, Education/Encouragement, Assessment, and Management/Monitoring of patients with dAEs to all anticancer therapies (Ciccolini, 2015). These principles allow for the holistic care of this patient population, and we postulate that advanced care providers and nurses using the CREAM principles will enhance the quality of life related to dAEs and irdAEs.

Advanced care providers are positioned to play an integral role in the oncology setting, and numerous studies have shown that the role has a positive impact in access to care, patient quality of life, cost of care, and clinical excellence (Dyar, Lesperance, Shannon, Sloan, & Colon-Otero, 2012; Mason, DeRubeis, Foster, Taylor, & Worden, 2013; Oncology Nursing Society Board of Directors, 2015). Moreover, advanced care providers and nurses are also at the front line of care to initiate teaching and timely referrals for patients on immunotherapies. Lastly, it is important for advanced care providers and nurses to establish and maintain efficient communication patterns among multidisciplinary services and with patients to enhance the continuity of care and efficiency flow of clinical information.

**Treatment Algorithm**

The adapted treatment algorithm for advanced care providers and nurses features severity assessments and interventions for maculopapular rash, pruritus, and vitiligo caused by anti–PD-1 and CTLA-4 inhibitors (Table). Gentle skin care and sun-protective measures should be instituted for all patients starting such therapy. Topical steroids are the mainstay in treating maculopapular rash, pruritus (Belum et al., 2016; Fischer et al., 2013; Naidoo et al., 2015), and vitiligo (Belum et al., 2016) due to the pleiotropic anti-inflammatory and immunosuppressive effects.

**Table 1:**
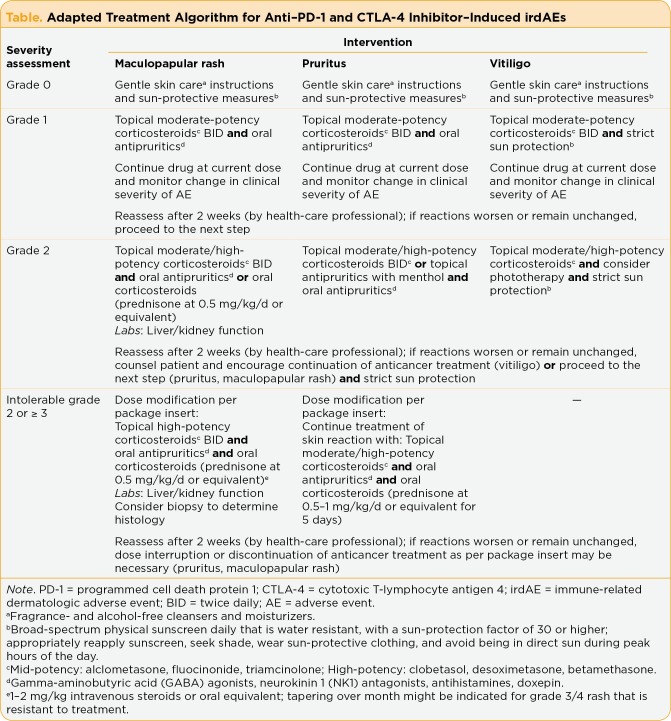
Adapted Treatment Algorithm for Anti–PD-1 and CTLA-4 Inhibitor–Induced irdAEs

General recommendations are to start with moderate-potency topical steroids, applying a thin layer twice daily for up to 1 month with clinical follow-up. For grade 1 irdAEs, patients generally continue the drug at the current dose and are monitored for clinical severity at follow-up after a 2-week period. If reactions worsen or remain unchanged, topical and oral therapy can be instituted for maculopapular rashes and pruritus. Treatment can include topical steroids, oral antipruritics, and oral corticosteroids. Patients should be reassessed after 2 weeks to encourage continuation of cancer treatment if possible. For intolerable grade 2 or 3 events, a skin biopsy and laboratory testing can be useful diagnostic tools; however, they may not lead to a conclusive diagnosis (Belum et al., 2016; Fischer et al., 2013; Naidoo et al., 2015).

In addition to a thorough skin and mucosal examination, laboratory testing can include renal and hepatic function (Bernstein et al., 2010; Naidoo et al., 2015). Finally, patients should be evaluated for infections and risks of developing infections in areas of rash and skin breakdown. Treatment should be based on culture and sensitivity results and reassessed at the start and end of antimicrobial therapy (Balagula, Lacouture, & Ito, 2014).

## CONCLUSION

Immunotherapies continue to provide a platform for improvements in clinical outcomes. The trend toward a multifaceted approach (i.e., combination therapies) will require expertise from all clinical teams, as irdAEs will continue to require attention and likely become more complex. Advanced care providers and nurses are integral players in the interdisciplinary oncodermatologic team for management of irdAEs caused by immunotherapies and promotion of minimizing dose modifications of treatment. Through timely dermatologic referrals, advanced care providers and nurses can advocate for treatment continuation by diligently preventing, managing, and monitoring irdAEs. Thus, facilitating for baseline, ongoing dermatologic assessments, and accurate grading is vital in the early prevention and therapeutic management of progressing adverse events.
